# O4.1. GENETIC VULNERABILITY TO DUSP22 PROMOTOR HYPERMETHYLATION IS INVOLVED IN THE RELATION BETWEEN IN UTERO FAMINE EXPOSURE AND SCHIZOPHRENIA

**DOI:** 10.1093/schbul/sby015.207

**Published:** 2018-04-01

**Authors:** Marco Boks, Lotte Houtepen, Zhida Xu, Yujie He, Gianluca Ursini, Adam Maihofer, Prashanth Rajarajan, Hilleke Hulshoff Pol, Bart Rutten, Andrew E Jaffe, Joel E Kleinmann, Dewleen Baker, Elly Hol, Schahram Akbarian, Caroline Nievergelt, Lot D Witte, Christiaan Vinkers, Daniel R Weinberger, Yaqin Yu, René Kahn

**Affiliations:** 1 University Medical Centre Utrecht; 2 Lieber Institute for Brain Development; 3 University of California, San Diego; 4 Icahn School of Medicine at Mount Sinai; 5 Rudolf Magnus Institute of Neurosciences, University Medical Center Utrecht; 6 Maastricht University Medical Centre; 7 Lieber Institute for Brain Development, Johns Hopkins Medical Campus; 8 Rudolf Magnus Center; 9 Brain Center Rudolf Magnus, University Medical Center Utrecht; 10 School of Public Health, Jilin University

## Abstract

**Background:**

Epigenetic changes may account for the doubled risk to develop schizophrenia in individuals exposed to famine in utero.

**Methods:**

We therefore investigated DNA methylation in a unique sample of patients and healthy individuals conceived during the great famine in China. To further examine the causality of the identified DNA methylation differences we also exposed human fibroblasts to nutritional deprivation and analyzed changes in expression and DNA methylation.

**Results:**

In the famine exposed schizophrenia patients we found significant hypermethylation of the dual specificity phosphatase 22 (DUSP22) gene promoter (Chr6:291687–293285) (N=153, p=0.01). The presence of a direct link between famine exposure and DUSP22 transcription was supported by increased methylation (p=0.048) and expression (p=0.019) in response to nutritional deprivation in the cultured human fibroblasts (N=10). These findings are in line with previous research that implicated hypermethylation of DUSP22 in the environmental risk to neuropsychiatric disorders. In postmortem brain samples from schizophrenia patients, variation in DUSP22 methylation was genetically regulated across chromosomes by a region on chromosome 16. This cross chromosomal regulation of variability in DUSP22 methylation is consistent with new 3D genome interaction data obtained using Hi-C capture in brain and previously published data on lymphocytes.

**Discussion:**

Together our results identify an epigenetic locus at which the response to prenatal famine exposure is genetically regulated across chromosomes and that is relevant for a major psychiatric disorder.

